# Simultaneous electroporation and dielectrophoresis in non-electrolytic micro/nano-electroporation

**DOI:** 10.1038/s41598-018-20535-6

**Published:** 2018-02-06

**Authors:** Chenang Lyu, Jianping Wang, Matthew Powell-Palm, Boris Rubinsky

**Affiliations:** 1 Zhejiang University, College of Biosystems Engineering and Food Science, Hangzhou, 310058 China; 2 University of California Berkeley, Department of Mechanical Engineering, Berkeley, CA 94720 USA

## Abstract

It was recently shown that electrolysis may play a substantial detrimental role in microfluidic electroporation. To overcome this problem, we have developed a non-electrolytic micro/nano electroporation (NEME) electrode surface, in which the metal electrodes are coated with a dielectric. A COMSOL based numerical scheme was used to simultaneously calculate the excitation frequency and dielectric material properties dependent electric field delivered across the dielectric, fluid flow, electroporation field and Clausius-Mossotti factor for yeast and *E. coli* cells flowing in a channel flow across a NEME surface. A two-layer model for yeast and a three-layer model for *E. coli* was used. The numerical analysis shows that in NEME electroporation, the electric fields could induce electroporation and dielectrophoresis simultaneously. The simultaneous occurrence of electroporation and dielectrophoresis gives rise to several interesting phenomena. For example, we found that a certain frequency exists for which an intact yeast cell is drawn to the NEME electrode, and once electroporated, the yeast cell is pushed back in the bulk fluid. The results suggest that developing electroporation technologies that combine, simultaneously, electroporation and dielectrophoresis could lead to new applications. Obviously, this is an early stage numerical study and much more theoretical and experimental research is needed.

## Introduction

Electroporation is the permeabilization of the cell membrane in response to the application of certain electric fields across the membrane, which can be reversible or irreversible^1–5^. In reversible electroporation, the cell membrane reverts to its impermeable state when the effect of the applied electric field concludes, while in irreversible electroporation, the cell succumbs to the electroporation process and dies. Both reversible and irreversible electroporation have become of importance to medicine and biomedical technologies, with applications ranging from ablation of cells^6^ to gene transfection^7^, nanomedicine^8^, CRISPR manipulation^9^, and many others.

The advent of micro/nano-electromechanical (MEMS) technologies has led to the development of single cell micro-electroporation devices^10–16^. Today, single-cell level technology is at the frontier of biomedical research. Working in the field of MEMS based electroporation devices for over two decades^10,17^, our group has developed, several generations of microelectroporation technologies, e.g^18–21^. Experiments with our more recent devices have shown that in micro and nanoscale electroporation devices, electrolysis occurs at the electrodes, simultaneously with electroporation^22^. In many situations, the effects of electrolysis obfuscate the effects of electroporation^23,24^. It was suggested that it may be desirable to eliminate electrolysis during electroporation^25^, in particular in gene transfection^26–28^.

**Figure 1:**
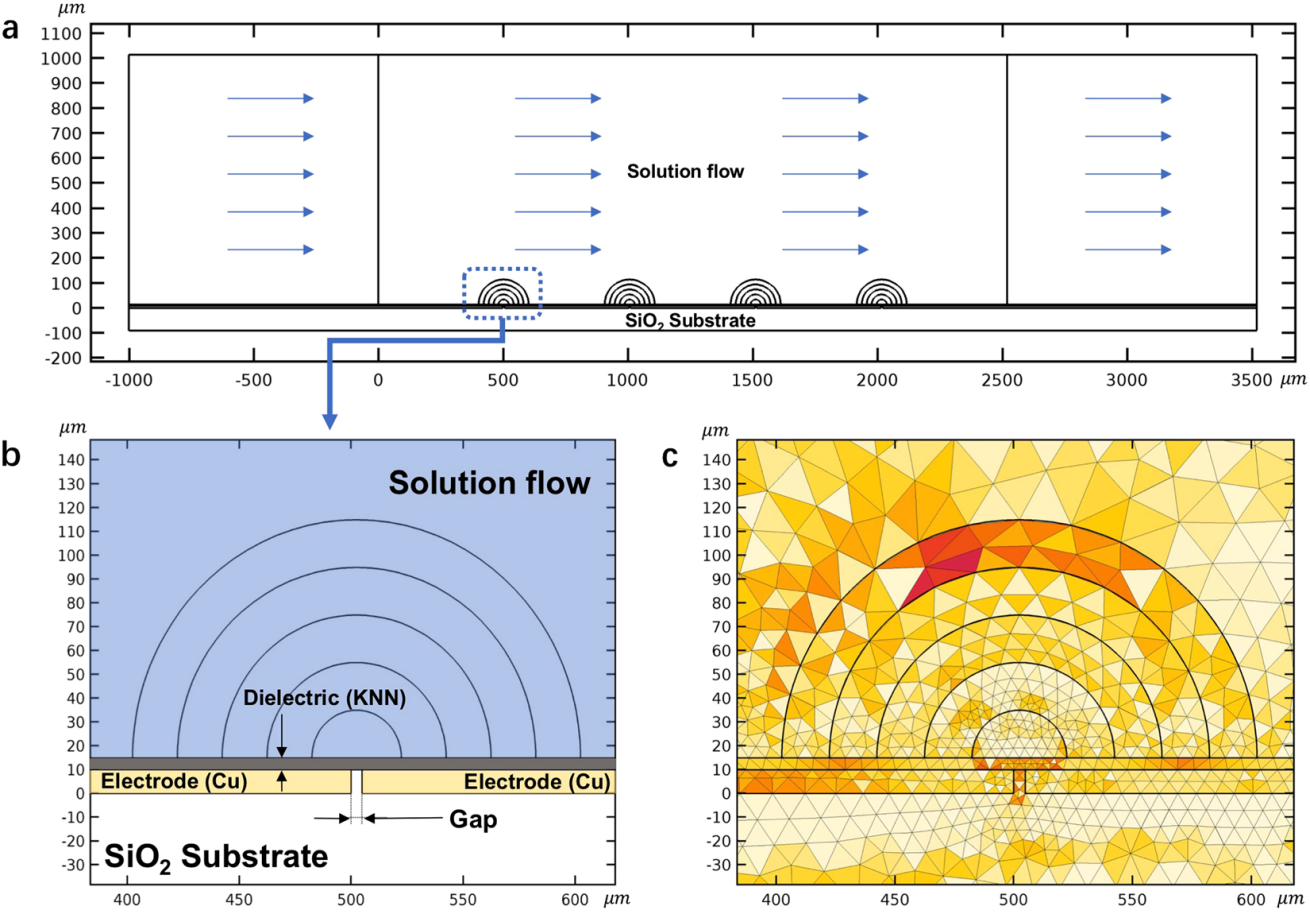
**(a**) The sectional schematic of the (NEME) non-electrolytic micro/nano electroporation (NEME) device. It is formed by a succession of electrodes, each separated from the other by an infinitesimal gap and coated with a dielectric. A solution containing cells flows through the channel upon the surface of the dielectric. **(b)** A magnified detail of the NEME surface which insulates between the electrodes. **(c)** The mesh (triangle) distribution near the gap. Concentric semi-circles were added to obtain a finer mesh (The mesh distribution without the concentric rings near the gap was shown in Supplementary Fig. S1). The figure was drawn based on COMSOL Multiphysics 4.3.

Mathematical modeling and experiments alike have shown that a possible way to reduce electrolytic effects at the micro and nano scale is to deliver the electroporation fields using alternating currents (AC)^19^. However, this method does not eliminate electrolysis in its entirety. Therefore, to completely eliminate electrolysis, we have designed a non-electrolytic micro/nano-electroporation (NEME) surface, in which the electroporation inducing electric fields are delivered to the target medium across a dielectric, with capacitive coupling^22,29^. Figure 1a and b shows a sectional schematic of a typical NEME surface. It is formed by a succession of interdigital electrodes, each separated from the other by a small insulating gap. The electrodes are coated with a dielectric, to eliminate electrolysis. This design requires the use of AC electric fields for electroporation. The use of AC electric fields in electroporation is not new^19,30–32^. The novelty in our design is the delivery of the AC electroporation fields across a composite surface, comprised of a sequence of electrodes separated by an insulating gap, coated with a dielectric, to eliminate electrolysis. The electrodes in previous studies on AC electroporation are in direct contact with the fluid and electrolysis occurs at the electrodes.

While analyzing the “electric fields” generated for AC electroporation on the NEME surface described above^29^, we have observed that the attendant “electric field gradients” are reminiscent of those used in many microfluidic devices, for dielectrophoresis^33–39^. This raised the possibility that, while eliminating electrolysis during electroporation, our new non-electrolytic electroporation device may cause the simultaneous manifestation of dielectrophoresis and electroporation. The consequences of the simultaneous occurrence of the dielectrophoresis and electroporation combination are unknown, and the goal of this paper is to elucidate the phenomenon. (Briefly, dielectrophoresis (DEP) is a phenomenon that occurs when a gradient of electric fields exerts a force on a dielectric particle^40,41^. The strength and direction of the force are affected by various factors such as the permittivity, shape, and size of the particle, the permittivity of the particle surrounding media, the electromagnetic field frequency, and the fluid mechanics of the medium. Because different cell/surrounding media combinations have various dielectrophoretic properties, dielectrophoresis can be used for separation of cells^42–48^).

Several studies in which electroporation and dielectrophoresis are discussed in the same paper have been published in the technical literature. These studies of two types: a) Evaluation of dielectrophoretic properties of cells before and after electroporation and b) using dielectrophoresis to process cells before or after electroporation. The number of studies of this type is rather large, and we will bring here only a few examples for illustration purpose. In a 2007 paper, Oblak *et al*.^49^ delivered typical millisecond long pulsed high electric fields across mouse melanoma cells in an electroporation device and then probed the dielectrophoretic properties of electroporated and non-electroporated cells in an interdigital electrode device with frequencies between 5 kHz and 50 MHz. Experimental results show that the electroporated cells have a different dielectrophoretic frequency dependent behavior from the non-electroporated cells. In 2008, Sedgwick *et al*.^35^ introduced a single cell micro electroporation device in which dielectrophoresis induced by oscillatory electric fields with a frequency of about 1 MHz is used to bring a cell close to an electroporation microelectrode, and following which, (sequentially), a different sinusoidal electric field (frequencies on the order of 0.1 MHz) are used to irreversibly electroporate the cells. In a 2013 paper, Salimi *et al*.^36^ describe a microfluidic device with interdigital electrodes in which high electric field pulses (4 pulses, 3 kV/cm, 100 $\mu s$ long) were delivered first to induce electroporation in cells and then 0.1 MHz to 10 MHz frequencies are applied to affect the treated cells location in the microfluidic device by dielectrophoresis; for detection with microwave frequency readings. The 2013 study of Moisescu *et al*.^38^ examines the dielectrophoretic properties of murine melanoma cells, electroporated in an electroporation chamber with either pulsed electric fields or with exponential decay pulses. Dielectrophoresis, done in a different device shows that the electroporated cells can develop different dielectrophoretic properties from non-electroporated cells. In a 2014 paper, Wei *et al*.^39^ describe a new flow-through microfluidic device made of three segments. The first segment employs pulsed electric fields to induce electroporation, followed by a segment in which sinusoidal electric fields were used to separate between the live and dead cells following electroporation. The last segment was used to collect separately living and dead cells. In a 2017 paper, Salimi *et al*.^50^ used a similar concept to that in their 2013 paper in which electroporation is induced first with pulsed electric fields followed by sinusoidal field excitations to analyze the electroporated cells with dielectrophoresis. These are just a few typical studies in papers that deal with both electroporation and dielectrophoresis.

A thorough review of papers involving both electroporation and dielectrophoresis, show two main attributes of the past research. First, all the research done in the past reached the conclusion that electroporation modifies the dielectrophoretic behavior of the treated cells. Second, in all the published research that we have examined, the electrical excitations for producing electroporation and dielectrophoresis are delivered separately; the phenomena of electroporation and dielectrophoresis occur sequentially. In contrast, in our NEME surface based devices, the phenomena of electroporation and dielectrophoresis occur simultaneously. Furthermore, once electroporation occurs and the dielectrophoretic properties of cells change, changes also occur in the way the dielectrophoretic forces affect the treated cells. To the best of our knowledge, there is no published study in which the phenomena of electroporation and dielectrophoresis are examined simultaneously and their reciprocal effects studied. Such a study is important for characterization of the NEME surface. To this end we performed numerical experiments using COMSOL, to evaluate the possible scenario when sinusoidal oscillatory electric fields simultaneously cause electroporation and dielectrophoresis. The numerical experiments were performed on models of two cell types, *E. coli* and yeast in a flow-through configuration. Obviously, this paper is only the start of the research on NEME surface based devices. Substantial additional work and in particular experimental work is needed to make the proposed technology practical.

## Results and Discussion

This work reports results from numerical simulations using COMSOL, on the behavior of cells flowing over a NEME surface, designed to induce non-electrolytic electroporation, by applying sinusoidal electric fields across a dielectric. The study solves for the electrical field in a fluid flowing with uniform laminar flow across the NEME surface. The calculated electric fields are used to assess electroporation of cells in the fluid. In addition, combining the calculated electric fields with models of cells, we evaluate the dielectrophoretic forces on cells. These calculations are done simultaneously to try and elucidate the phenomena which occur in a system in which cells flowing across a NEME surface experience simultaneously, electroporation and dielectrophoresis. Of particular relevance to this study is the following property of dielectrophoresis: the force exerted on a cell depends on the relative polarizability of the cell and that of the surrounding medium. Dependent on the relation between these polarizabilities the cell can move in the direction of diverging electric fields or in the direction of converging electric fields. When a cell moves in the direction of increasing electric fields, this is known as positive DEP (pDEP) and when the cell moves in the direction of decreasing electric fields, this is known as negative DEP (nDEP). Because in this study we deal with radio-frequency-range alternating currents (AC) electric fields, delivered across a dielectric, we have ignored the effects of iontophoresis and electrophoresis. The use of sinusoidal electric fields allows the elimination of electrophoretic motion of particles due to inherent particle charge. The cells studied are *E. coli* and yeast. These microorganisms have been studied extensively and were chosen because their electrical property data for electroporated and non-electroporated cells is available. These microorganisms are of particular interest due to their importance in transgenic manipulation for biopharmaceutical applications and for sterilization.

**Figure 2:**
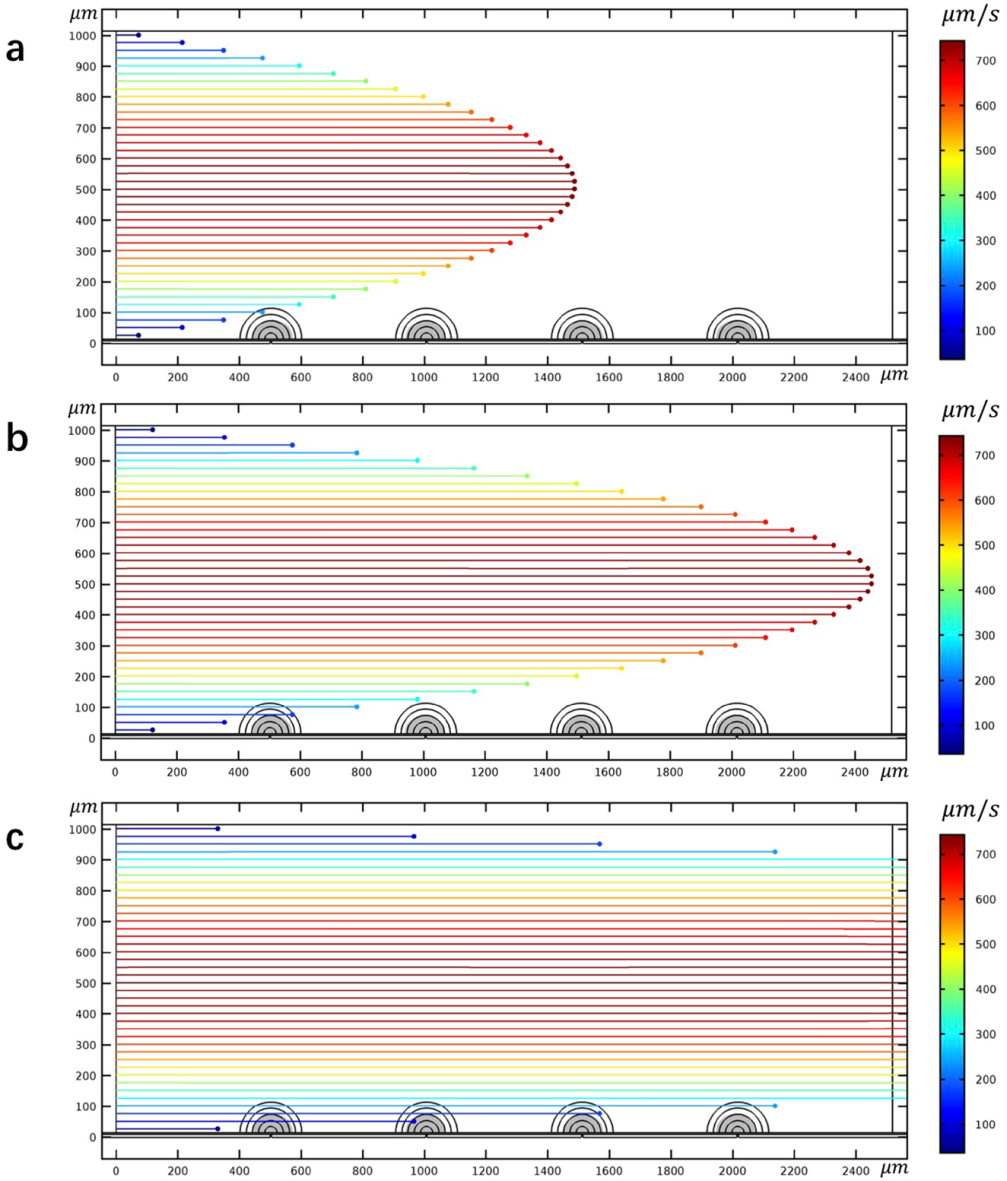
The trace lines of yeast at 2 s **(a)**, 3.3 s **(b)** and 9 s **(c)** when the dielectrophoretic effects of the electroporation fields are ignored. The electric field contours produced by the dielectric coated electrodes is also shown. Note that the streamlines are parallel to the surface and only particles very close to the surface will experience electroporation type fields. The figure was drawn based on COMSOL Multiphysics 4.3.

**Figure 3:**
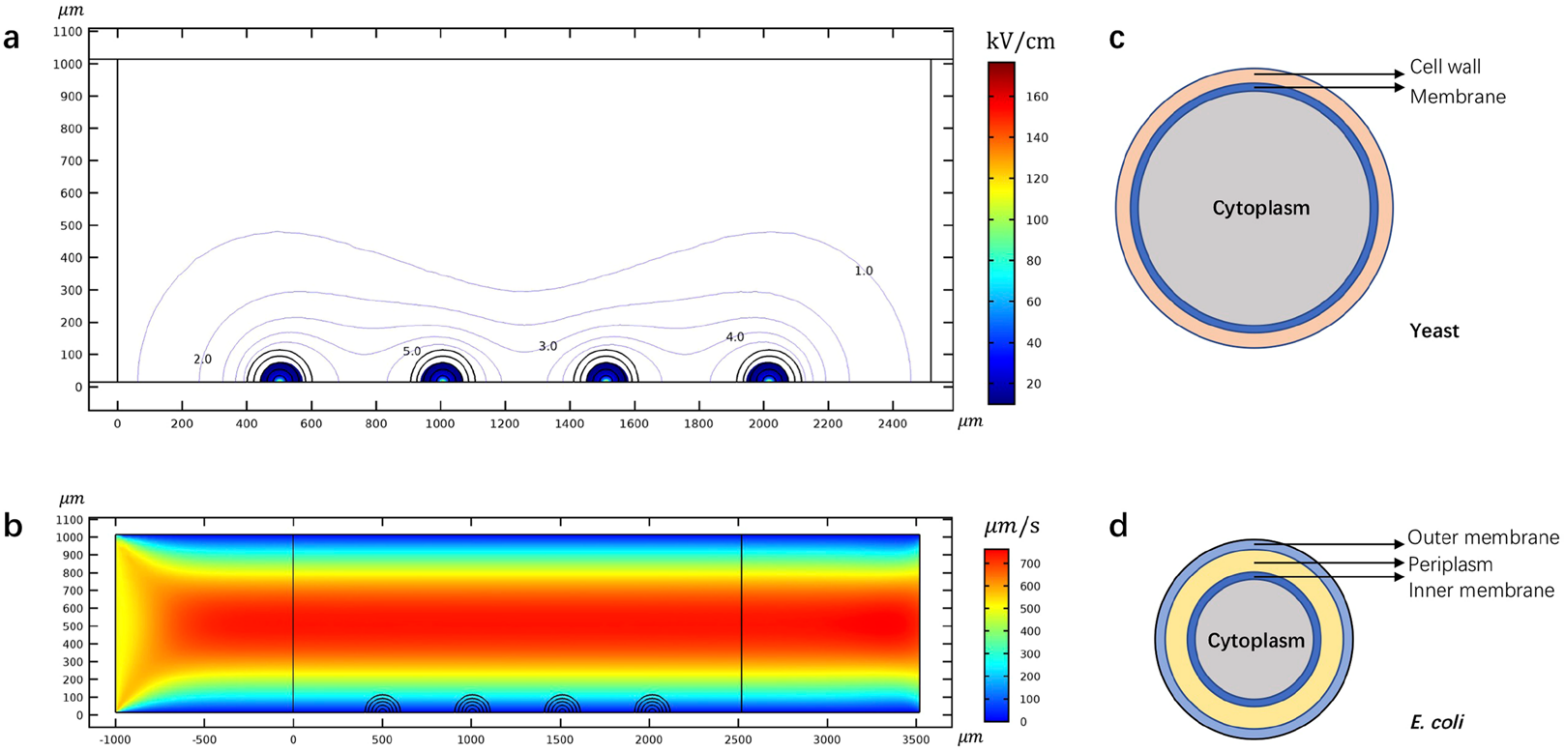
**(a)** A contour plot of the electric field distribution around the point of singularity. Values are given in kV/cm. The electric field is larger than 10 kV/cm within the blue area. **(b)** The map of fluid flow velocity in the channel. **(c)** Two-shell model of the yeast (not to scale) **(d)** Three-shell model of the *E. coli* (not to scale). The figure was drawn based on COMSOL Multiphysics 4.3.

First, to establish a baseline, we performed a numerical simulation of flow and electric fields across a NEME surface, that ignores dielectrophoresis. The results are given in Fig. 2a–c. These results are similar to all previous studies of micro/nano electroporation^13,51^. The calculations assume flow between two parallel plates, separated by 1 mm of a fluid with a viscosity of 10^−3^ Pa*s, density of 10^3^ kg/m^3^. The flow is laminar and uniform with inlet flow velocity of 500 μm/s. The electrical boundary conditions on the NEME surface are a peak-to-peak voltage of 100 V delivered at a frequency of 2.5 × 10^5^ Hz. Particles (yeast) were uniformly injected at the left side inlet of the channel. The particles were tracked in time and a sequence is shown in Fig. 2a–c. The figures show the stream lines of the particles 2.0 s, 3.3 s, and 9 s after the particles were injected at the channel inlet. The electric fields produced by the NEME surface electrodes are also shown in these figures. A higher magnification of the electric fields in Fig. 2 is given by Fig. 3a. It shows a typical electric field distribution in the fluid near the NEME electrodes surface. In NEME electroporation, the electric fields decrease radially from the insulating interface between two electrodes, similar to the electric fields around a dipole. These results are also similar to those in our previous studies^20,21^. The particles are carried by the flow and their track is parallel to the walls of the channel. It is seen that only the particles immediately adjacent to the wall will enter the region in which the electric fields are sufficient to produce electroporation. It is important to note, for the coming discussion, that the depth of penetration of the field in the NEME configuration discussed here is limited. This was one of the drawbacks of our original NEME technology. While the voltages needed for NEME are small, the depth of penetration of the electric field is shallow and only cells very close to the NEME surface can be electroporated. Therefore, this configuration can effectively electroporate cells in a microchannel^51^, however, it cannot affect most cells in a macro channel, like the one shown in Fig. 2a–c. It is evident that in a macro channel, only the cells close to the NEME surface will be electroporated, while those further away will not.

**Figure 4:**
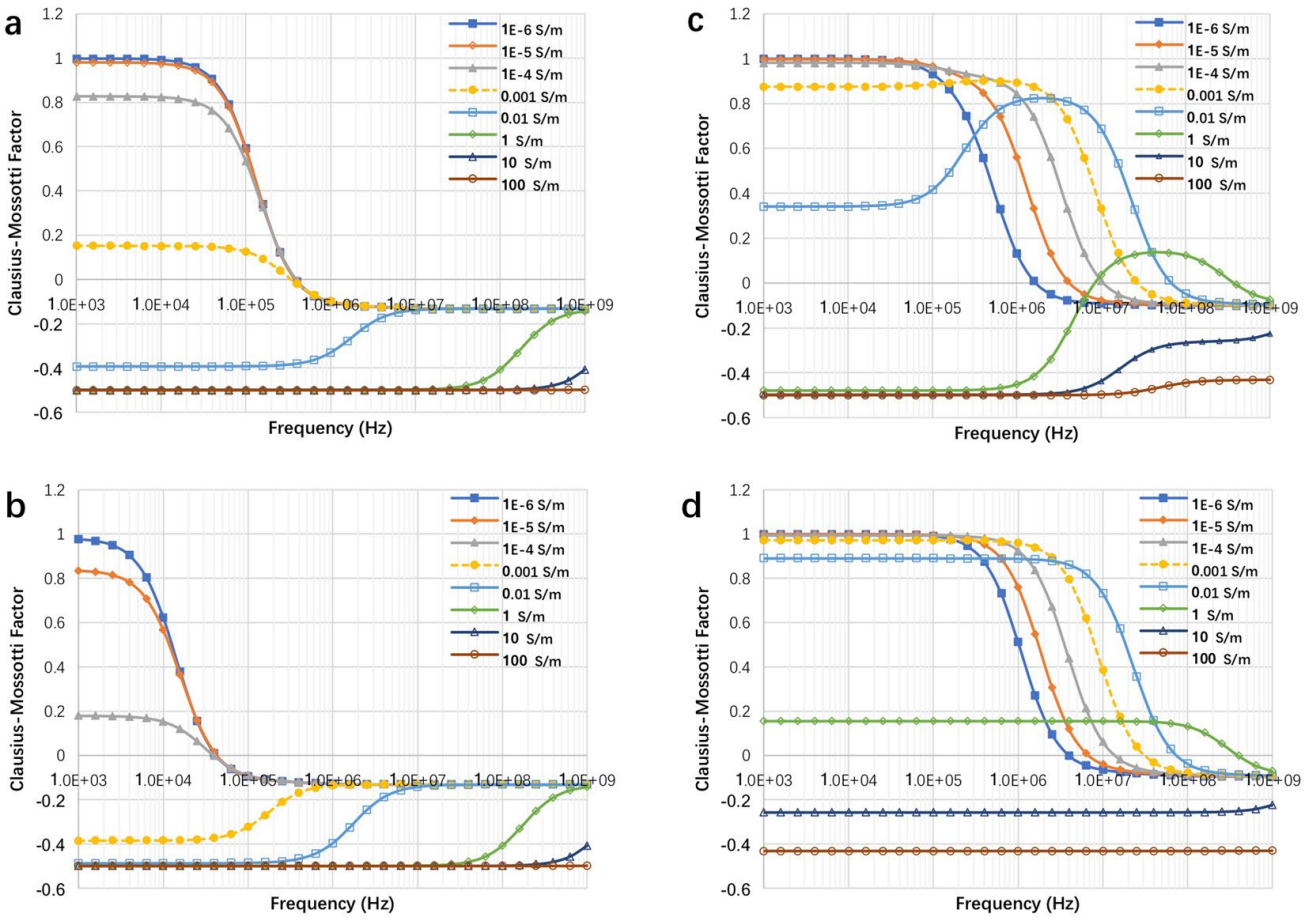
The Clausius-Mossotti factor of live yeast **(c)**, dead yeast **(d)**, live *E. coli* **(e)**, electroporated *E. coli* **(f)** in the function of frequency with different surrounding solution conductivity. The figure was drawn based on COMSOL Multiphysics 4.3.

The Clausius-Mossotti (CM) factor is a key parameter in dielectrophoresis modeling. It provides a measure of the dielectrophoretic forces on the particles. We have calculated the CM factor for live and dead yeast using the two-shell model^52^ in Fig. 3c. For live and electroporated *E. coli* we used the three-shell model of Hölzel^53^ (Fig. 3d**)**. The model of Hölzel is based on experimental data. The *E. coli* is rod-shaped and using a more precise geometrical simulation of the rod shape, such as an oblate ellipsoid^54^ could improve the hydrodynamic calculations. Using the simpler spherical model may reduce the accuracy of the model, with regards to the effects of fluid flow on the motion of the particle. However, in this study, the hydrodynamic calculations assume that the particle has a similar density to the fluid and therefore it will act, with respect to hydrodynamic forces, as a particle following the stream lines. The focus of this study is to investigate the effects of dielectrophoresis in a NEME device. The effects of dielectrophoresis are modeled better by the experimentally validated three-shell model of Hölzel^53^. The electrical and physical parameters used are listed in Table 1. The CM factor was calculated for a range of frequencies of interest and for a range of electrical properties of the fluid media surrounding the cell. The results are shown in Fig. 4. Obviously, the CM factor is strongly dependent on the frequency and the electrical properties of the surrounding medium. Furthermore, there is a distinct difference between each of the four panels, for the different cell types and physiological condition of the cells. On this fact rests the principle of cell separation by dielectrophoresis. It is important to notice that in each of the curves, in all four panels, there is a frequency at which a transition occurs from a positive CM factor to a negative CM factor, which represents the transition from pDEP to nDEP. This is also an important element in the use of dielectrophoresis for effective separation between particles (cells) of different types.

**Table 1:** The electrical properties of the dielectrics and cells used in this study.

Name	Value	Description			
Solution and Dielectric^29^					
${\sigma }_{f}$	1E-3 S/m	The conductivity of solution (Saline solution)			
${\varepsilon }_{r,f}$	78	The relative permittivity of solution (Saline solution)			
${\sigma }_{i}$	1E-10 S/m	The conductivity of dielectric (Sodium Potassium Niobate)			
${\varepsilon }_{r,i}$	750	The relative permittivity of dielectric (Sodium Potassium Niobate)			
**Name**	**Value**	**Description**	**Name**	**Value**	**Description**
**Yeast** ^57^			***E. coli*** ^53,58^		
${r}_{y}$	4 $\mu m$	Yeast’s radius	${r}_{e}$	2 $\mu m$	*E. coli* radius
${\sigma }_{p\text{\_}y}$	0.2 S/m	Cytoplasm’s conductivity	${\sigma }_{p\_e}$	0.22 S/m	Cytoplasm’s conductivity
${\varepsilon }_{r,p\_y}$	50	Cytoplasm’s relative permittivity	${\varepsilon }_{r,p\_e}$	60	Cytoplasm’s relative permittivity
${\sigma }_{s1\_y}$	2.5E-7 S/m	Membrane’s conductivity	${\sigma }_{s1\_e}$	1E-6 S/m	Inner membrane’s conductivity
${\varepsilon }_{r,s1\_y}$	6	Membrane’s relative permittivity	${\varepsilon }_{r,s1\_e}$	5.5	Inner membrane’s relative permittivity
$t{h}_{s1\_y}$	8 nm	Membrane’s thickness	$t{h}_{s1\_e}$	7 nm	Inner membrane’s thickness
${\sigma }_{s2\_y}$	1.4E-2 S/m	Cell wall’s conductivity	${\sigma }_{s2\_e}$	31* ${{\sigma }_{f}}^{0.4}\,$S/m	Periplasm’s conductivity
${\varepsilon }_{r,s2\_y}$	60	Cell wall’s relative permittivity	${\varepsilon }_{r,s2\_e}$	60	Periplasm’s relative permittivity
$t{h}_{s2\_y}$	220 nm	Cell wall’s thickness	$t{h}_{s2\_e}$	50 nm	Periplasm’s thickness
			${\sigma }_{s3\_e}$	1e-4 S/m	Outer membrane’s conductivity
			${\varepsilon }_{r,s3\_e}$	12	Outer membrane’s relative permittivity
			$t{h}_{s3\_e}$	7 nm	Outer membrane’s thickness
**Dead yeast** ^57^			**Electroporated** ***E. coli*** ^58,59^		
${\sigma }_{p\_dy}$	7E-3 S/m	Cytoplasm’s conductivity	${\sigma }_{p\_de}$	0.09 S/m	Cytoplasm’s conductivity
${\sigma }_{s1\_dy}$	1.6E-4 S/m	Membrane’s conductivity	${\sigma }_{s1\_de}$	1E-2 S/m	Inner membrane’s conductivity
${\sigma }_{s2\_dy}$	1.5E-3 S/m	Cell wall’s conductivity	${\sigma }_{s2\_de}$	31* ${{\sigma }_{f}}^{0.4}\,$S/m	Periplasm’s conductivity
			${\sigma }_{s3\_de}$	1 S/m	Outer membrane’s conductivity

**Figure 5:**
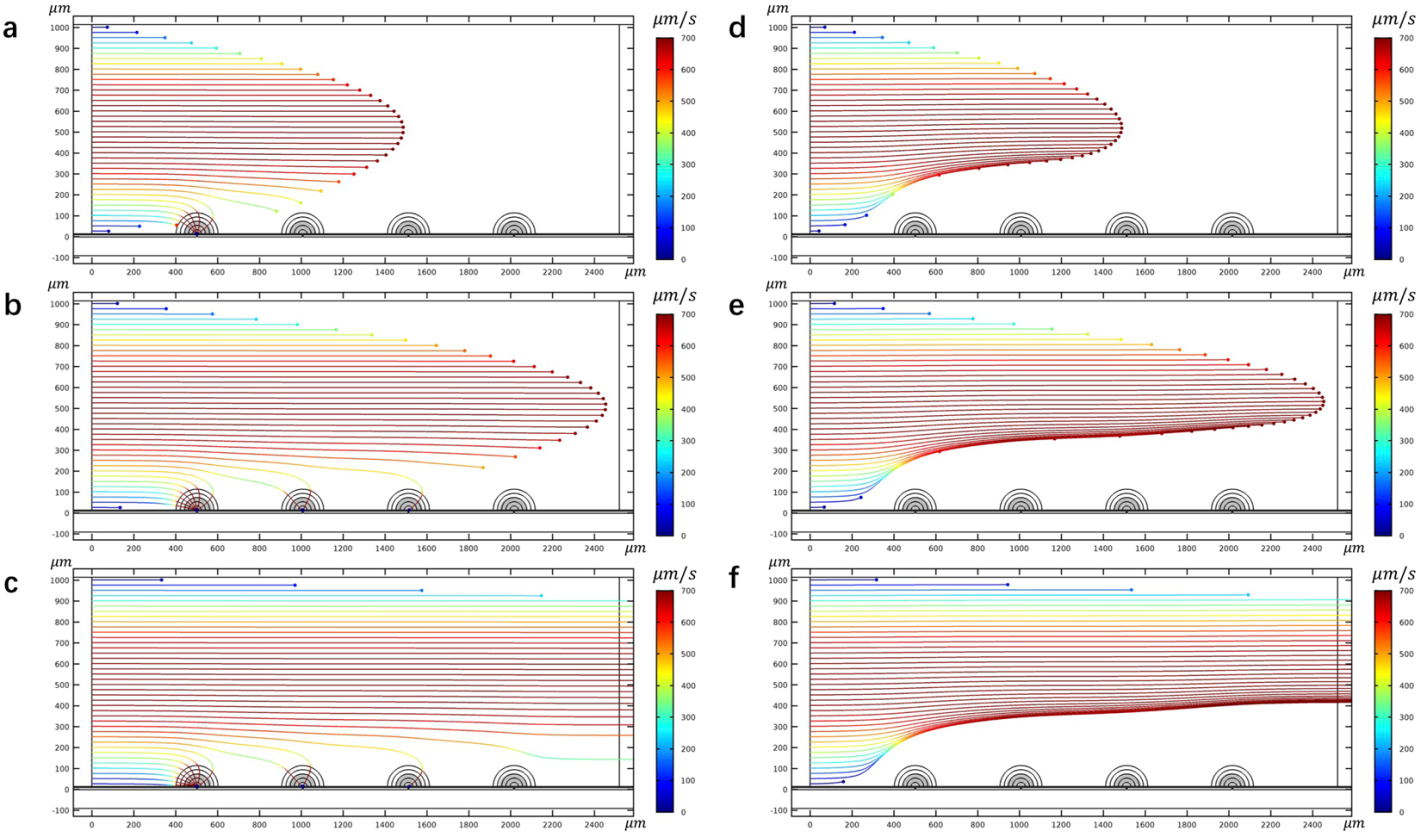
The trace lines of yeast at 2 s **(a)**, 3.3 s **(b)**, and 9 s **(c)** when the pDEP attractive the live yeasts. The trace lines of yeast at 2 s **(d)**, 3.3 s **(e)**, and 9 s **(f)** when the nDEP push away the dead yeasts. The figure was drawn based on COMSOL Multiphysics 4.3.

Figure 5 is included to illustrate the technique involved in the separation of different cell types by dielectrophoresis. The separation technology employs CM frequency and surrounding solution conductivity-dependent curves, like those in Fig. 4, to identify the parameters needed for separation between different cell types. For example, Fig. 4 shows that in a fluid with a conductivity of 10^−3^ S/m (typical to tap water) and at a frequency of 2.5 × 10^5^ Hz, the CM factor is positive for the live yeast and negative for the dead yeast. These parameters can be used to separate between live and dead yeast by dielectrophoresis and collect the live yeast on the electrode surface. For example, Fig. 5d–f follow the track of irreversible electroporated yeast transport in time when a frequency of 2.5 × 10^5^ Hz and a voltage of 100 V is applied between the NEME electroporation electrodes in a solution with a conductivity of 10^−3^ S/m. The electrical and fluid flow parameters are the same as those used to generate the results in Fig. 2. It is seen that here, nDEP affects the dead yeast cells and they are rejected from the electroporation surface. Figure 5a–c track the path of live yeast in time, when a similar frequency of 2.5 × 10^5^ Hz is applied between the NEME electroporation electrodes in a solution with a conductivity of 10^−3^ S/m. The electrical parameters are also the same as those used to generate the results in Fig. 2. It is seen that pDEP affects the live yeast, and they are attracted to the electroporation surface. This is how live and dead yeast can be separated by dielectrophoresis, outlining the basic principle by which dielectrophoresis is used for separation between different cell types^42,43^.

A comparison of Figs 2 and 5a to c provides insight into the consequences of modeling a process of NEME surface based electroporation, while ignoring the simultaneous effects of dielectrophoresis and electroporation. Figure 2 shows that when the phenomenon of dielectrophoresis is ignored, the cells follow the fluid streamlines. However, when the phenomenon of dielectrophoresis is included in the model, the cells can be attracted or rejected from the NEME electroporation surface. Obviously, this observation on the effect of sinusoidal electric fields is not new. However, it is brought here to show that it may have a substantial impact on cells electroporated by the NEME technology. The simultaneous occurrence of dielectrophoresis and electroporation could have a positive or negative effect on the electroporation. If the frequency is such that the cell experiences pDEP it will increase the number of cells electroporated. This is particularly valuable for electroporation in macro channels or macro chambers. However, if the frequency causes nDEP, the cells will fail to become electroporated. Therefore, when using the NEME technology for electroporation, consideration of the simultaneous occurrence of electroporation and dielectrophoresis becomes important.

**Figure 6:**
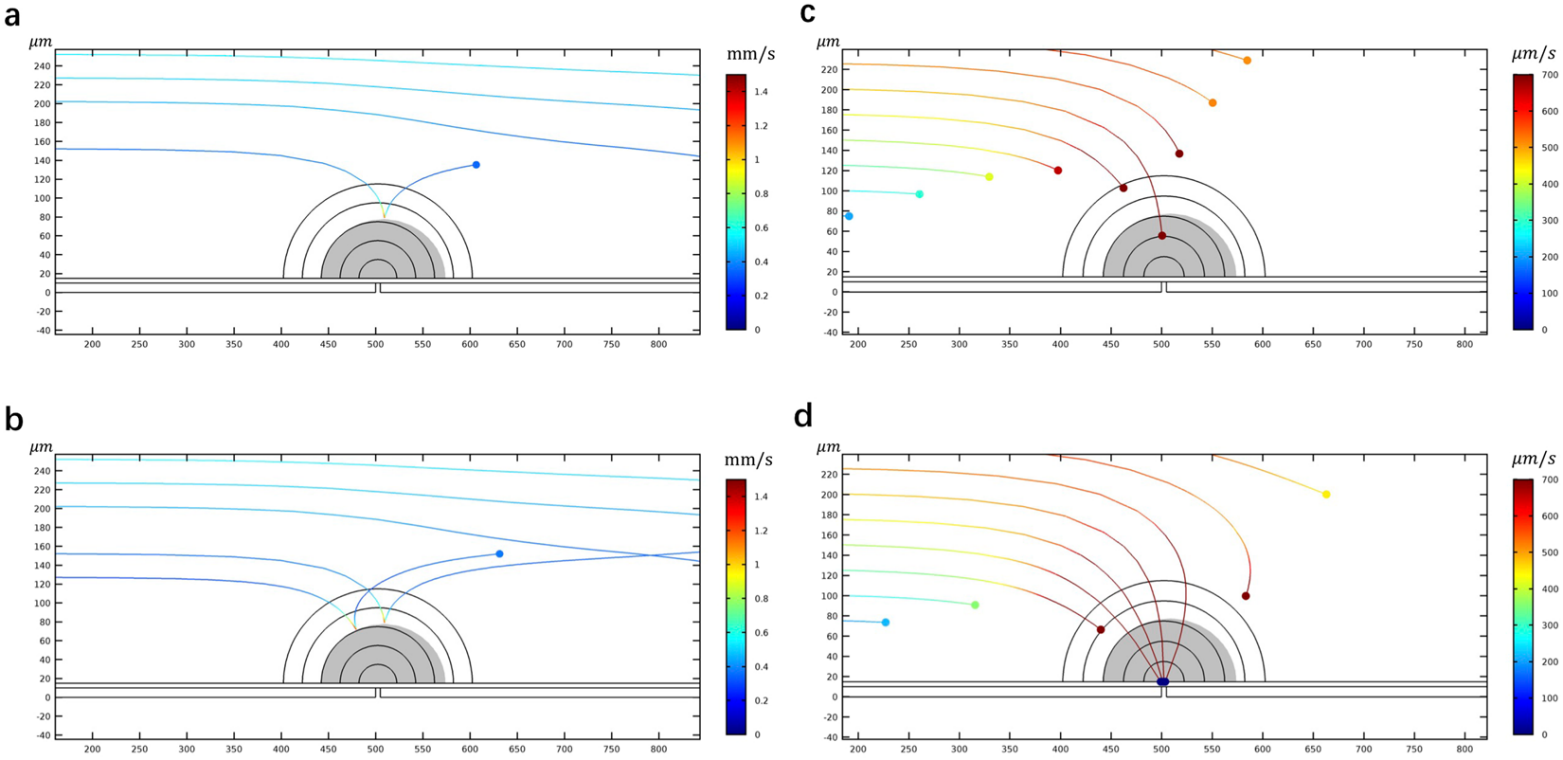
**(a,b)** The track of two live yeast cells which experience first pDEP (attracted to the NEME electrodes) and then are killed by irreversible electroporation, after which they experience nDEP (rejection from the NEME electrodes). In this model, the frequency is 2.5 × 10^5^ Hz, the electrical conductivity of the solution is 10^−3^ S/m. **(c,d)** The track of several *E. coli* cells which experience pDEP and are attracted to the NEME electrodes surface. In this example, the frequency is 10^6^ Hz, and electrical conductivity of the solution is 10^−3^ S/m. With these parameters, the pDEP force acting on the cells actually becomes larger with electroporation and the *E. coli* will bind to the electrode surface. The figure was drawn based on COMSOL Multiphysics 4.3.

Figure 6 reveals new aspects of the combination of electroporation and dielectrophoresis, that may have practical importance for NEME technologies. Figure 4a and b, show that in the frequency range of between 4 × 10^4^ Hz and 4 × 10^5^ Hz when the fluid conductivity is smaller than 0.001 S/m, a live yeast cell experiences pDEP (attracted to the electrode) while a dead yeast cell experiences nDEP (rejected from the electrodes). We wanted to model what will happen when a cell is attracted to an NEME electroporation electrode, but in the process, it undergoes irreversible electroporation (i.e. it dies). We have measured the change in impedance of single cells during and after the application of electroporation pulses^55^. Measurements show that the change in impedance upon the application of electroporation inducing electric fields occurs within milliseconds^55^. In the case of reversible electroporation, the return to normal impedance occurs in many seconds to seconds. The time scale in which the impedance of the cell changes upon electroporation is shorter by orders of magnitude from the time scale of flows. Therefore, in this analysis, we assumed that in the time scale of the fluid flow, the dielectrophoretic properties of the cell will change as soon as it reaches an electric field that induces electroporation. Figure 6a and b track live yeast particles as they approach a NEME generated electric field. The figures show that at first, the live yeast will be attracted by the pDEP force to the electrode. The attracted cells are originally at a distance from the NEME electric field, where in the absence of dielectrophoresis the cells will be unaffected. However, as the dielectrophoresis force brings the yeast towards the NEME surface, the cells reach an electric field that can induce electroporation. In the example of Fig. 6a and b, the dark semicircles around the NEME represent the region in which the electric field is higher than 10 kV/cm. In our calculations, the cells reside in this area for several ms, which is sufficient to cause cell death by irreversible electroporation. If the AC field is such that irreversible electroporation can occur and the yeast dies, the CM factor changes from that for the live yeast to that for dead yeast.

When the NEME electric field frequency ranges between 4 × 10^4^ Hz and 4 × 10^5^ Hz and when the fluid conductivity is smaller than 0.001 S/m, pDEP will change into nDEP and the dead cell will be rejected into the fluid. This is significant to both the uses of sinusoidal voltage excitations for dielectrophoresis and electroporation. For example, dielectrophoresis is often used to separate and collect cells at electrodes. However, our study shows that there can be frequencies in which the cells driven towards the electrodes by dielectrophoresis will inadvertently undergo electroporation and will be rejected back into the bulk of the solution. This is what Fig. 6a and b shows. On the other hand, when a sinusoidal electric field is chosen to induce electroporation in cells, but the frequency is such that the cell experiences a nDEP force, the cell will not reach the vicinity of the electrodes and will not be electroporated. Our numerical analysis shows that when using sinusoidal electric fields with the goal of either electroporation or dielectrophoresis it is important to realize that both phenomena can occur simultaneously and the nature of the dependence is a strong function of the CM factor.

Realizing that dielectrophoresis and electroporation can occur simultaneously opens the door to new fields of application that take advantage of the simultaneous occurrence of both phenomena.

An important application of irreversible electroporation is sterilization of fluids. However, the electric fields required for sterilization are very high. When large volumes are sterilized by electroporation the voltages needed are very large, in the tens of kV, and the technology is massive and expensive. Furthermore, products of electrolysis contaminate the fluids. Micro- and nano irreversible electroporation were considered for sterilization because they require much lower voltage to induce electroporation^20,21^. However, as shown in Fig. 2c, the depth of penetration is small. Researchers sought other fluid flow configurations to bring the cells to the vicinity of the electrodes, to be irreversible electroporated^56^. However, the use of micro channels requires the use of high fluid pressure to overcome the flow resistance. We believe that using a NEME surface with AC fields can provide a new solution, that would work also in macro channels. Figure 6c and d show how combining dielectrophoresis and electroporation could be used for sterilization of large volumes with micro/nano electroporation devices. Using the CM factor diagram for dead and live *E. coli* in Fig. 4, it is possible to identify frequencies at which both live and dead *E. coli* experience pDEP. (Unlike the previous example in Fig. 6a and b in which we chose a frequency in which live yeast experienced pDEP and dead yeast experienced nDEP). Figure 6c and d show the track of several *E. coli* cells which experience pDEP and are attracted to the NEME electrodes surface. We have chosen to use here a frequency of 10^6^ Hz, and electrical conductivity of the solution of 10^−3^ S/m. With these parameters, the pDEP force acting on the cells actually becomes larger by electroporation and therefore, the force that attracts the *E. coli* to the NEME electrode surface will increase with electroporation. Figure 6c and d show that when dielectrophoresis is combined with electroporation, by choosing the frequency (10^6^ Hz) judiciously, the simultaneous combination attracts the *E. coli* to the electrodes where they stay.

**Figure 7:**
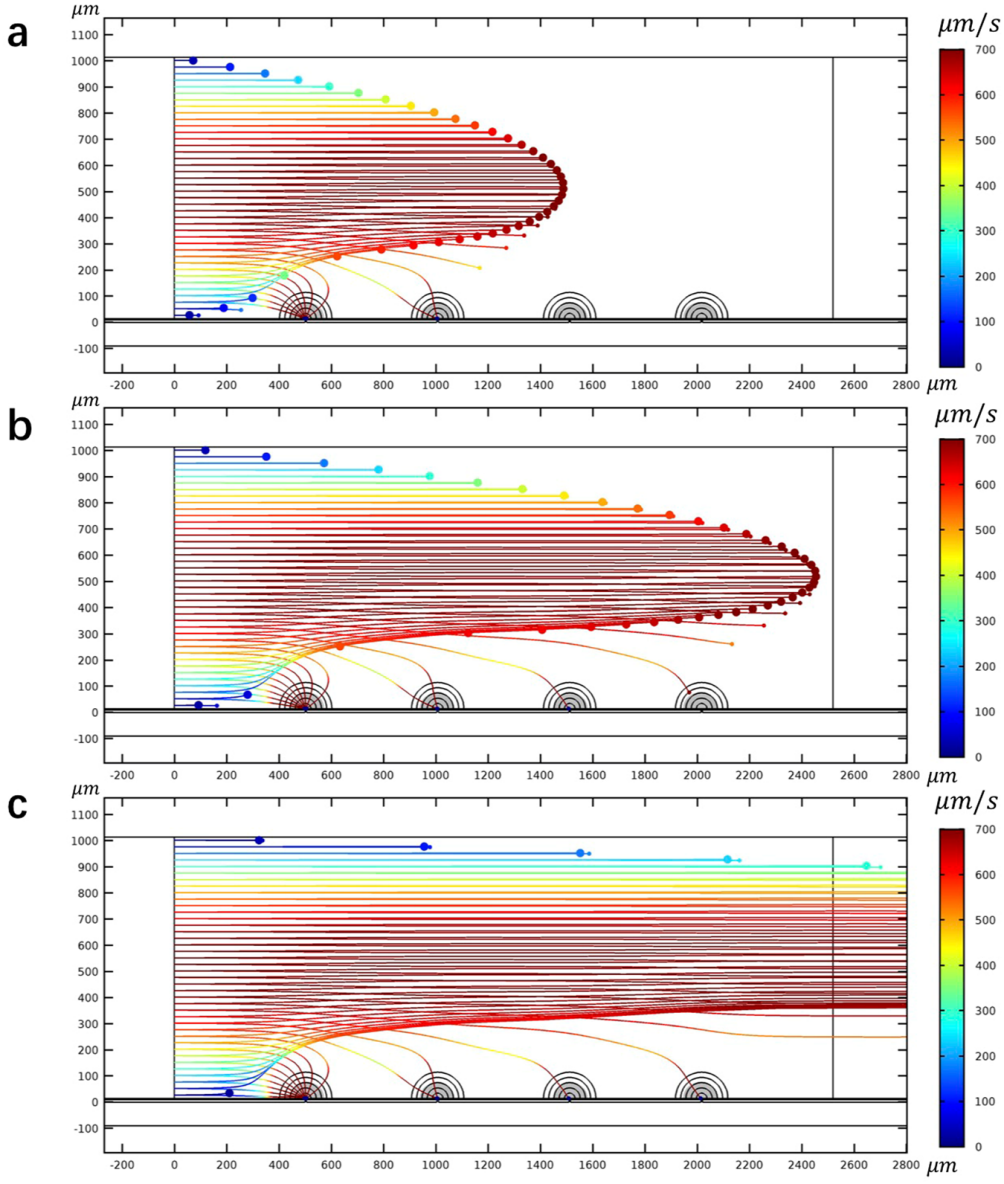
Tracking the yeast and *E. coli* flowing through a macro channel at 2 s **(a)**, 3.3 s **(b)** and 9 s **(c)**. The panels show that the *E. coli* cells (simulated by small diameter particles) are attracted to the NEME electrodes, where they are electroporated and killed, while the yeast cells (large diameter particles) are ejected by nDEP and flow through intact. The figure was drawn based on COMSOL Multiphysics 4.3.

Figure 7 presents another example that illustrates a possible use of the simultaneous dielectrophoresis/electroporation combination. Here we inject a population of yeast contaminated by *E. coli*. A frequency of 10^6^ Hz and a fluid conductivity of 0.001 S/m (dash line) were chosen from Fig. 2 to exert a nDEP force on live yeast, and a pDEP force on alive and dead *E. coli*. Tracking the cells in time, and simultaneously solving for dielectrophoresis and electroporation, Fig. 7 shows that the *E. coli* are attracted to the NEME surface, where it is electroporated and killed, while the yeast flows through intact. This presents but one of the applications that can be envisioned once it is realized that there are configurations in which dielectrophoresis can occur simultaneously with electroporation and that the effects can be controlled by controlling the AC frequency. This concept could be used, for further example, in a combination of irreversible electroporation and dielectrophoresis for sterilization of microorganisms from blood, or for removal and destruction of cancer cells in blood, or most generally for selective transfection of one type of cell from a group of varying cells.

## Conclusion

A numerical study was performed to study the phenomena which occur when cells flow across a non-electrolytic micro electroporation surface, activated by sinusoidal electric fields. COMSOL based calculations of: electric fields, fluid flow, dielectrophoresis forces, and electroporation effects on cells has shown that the phenomena of dielectrophoresis and electroporation can occur simultaneously in a non-electrolytic micro/nano-electroporation device, and that the outcome can be controlled by the applied sinusoidal voltage frequency. This simultaneous occurrence of dielectrophoresis and electroporation and the ability to control the outcome by choosing the desired frequency has many exciting applications in biotechnology and medicine, such as the separation of cells, destruction of unwanted cells, or the separation of cells and transfection of only one type of cells from a composite of varying cell types.

## Methods

The electrode geometry analyzed in this study is shown in Fig. 1a and b. The model is two-dimensional and consists of a succession of planar metal electrodes, separated by an insulating gap (point of singularity) and coated with a thin layer of a dielectric material, which replaces the galvanic coupling in conventional electroporation with a capacitive link. The overall schematic is shown in Fig. 1a. Displayed in Fig. 1b is a magnified detail of the point of singularity, which insulates between the electrodes. This active electrode surface may be viewed as a composite, micro/nano designed surface. To provide practical value to the analysis, we studied a specific practical design configuration that we recently developed^29^. It should be emphasized that the NEME concept is applicable to both micro and nano scale because the dimensions of the gap between the electrodes could be, in principle, infinitesimal. We chose this particular design here because in our previous studies we have gained substantial experience with these materials and parameters. The design consists of (10 μm) thickness copper electrodes, which rest on an amorphous SiO_2_ substrate. The copper electrodes are separated by a 5 μm gap. This gap size was originally chosen to obtain a larger electroporation area for the particular dielectric used^29^. A comparative study of different dielectrics has shown that a ceramic, Sodium Potassium Niobate (KNN)^29^, has electromagnetic properties that are optimal for a non-electrolytic micro-electroporation dielectric. The thickness of the dielectric “h_i_” in Fig. 1b is 5 μm. Obviously, other dimensions are possible, including nano scale gaps across the insulation between the NEME electrodes, depending on the desired application.

In the analysis, it is assumed that a solution containing cells flows in a channel flow, and the flow is incompressible with a no-slip boundary condition. To simulate a typical creeping flow, we set two infinite-like areas near the left inlet and right outlet of the channel (Fig. 3b). The cells were uniformly released at the left-hand side inlet where the stream lines are parallel. The NEME composite electrode structure lines the surface of the channel. Table 1 lists the electrical properties of the dielectrics, the solutions, and the cells used in this study.

Since our model contains both conductive (electrolyte containing solution) and dielectric materials (insulating coating of electrodes), both displacement and conduction currents exist. The governing equation is therefore, conservation of current:

1 $$\nabla \cdot \overrightarrow{{\rm{J}}}=0$$

where $\nabla \cdot (\,)$ is the divergence operator and $\overrightarrow{{\rm{J}}}$ stands for the local current density vector. The current density has a conductive component and a displacement component, and is given by:

2 $$\overrightarrow{{\rm{J}}}=({\rm{\sigma }}+{{\rm{\varepsilon }}}_{0}{{\rm{\varepsilon }}}_{{\rm{r}}}\frac{\partial }{\partial {\rm{t}}})\overrightarrow{{\rm{E}}}$$

$\overrightarrow{{\rm{E}}}$ represents the local electric field, ${\rm{\sigma }}$ is the conductivity, ${{\rm{\varepsilon }}}_{0}$ is the vacuum permittivity and ${{\rm{\varepsilon }}}_{{\rm{r}}}$ is the relative permittivity of the material. The electric field is linked to the potential field, U, by the relationship:

3 $$\overrightarrow{{\rm{E}}}=-\nabla {\rm{U}}$$

The field equation is solved for the geometrical configuration of Fig. 1a and b, subject to Dirichlet boundary condition (sinusoidal voltage), imposed at the electrodes. The remainder outer surface of the domain was insulated.

The fluid flow was assumed to be incompressible creeping flow, and the governing equations are:

4 $$\nabla \cdot [-{\rm{pI}}+{\rm{\mu }}(\nabla {\rm{u}}+{(\nabla {\rm{u}})}^{{\rm{T}}})]+{\rm{F}}=0$$

5 $${\rm{\rho }}\nabla \cdot ({\rm{u}})=0$$

with inlet flow conditions:

6 $${\rm{u}}=-{{\rm{U}}}_{0}{\rm{n}}$$

A two-shell model (Fig. 3a) was used to simulate the yeast cell^52^ and a three-shell model (Fig. 3b) was used to simulate the *E. coli* cell^54^ in the calculation of the DEP force of the particle. The cell models used in the study are using the electrical properties of yeast and *E. coli* (Table 1), two microorganisms of importance in biotechnology.

The DEP force of yeast (${{\rm{F}}}_{{\rm{y}}}$) and *E. coli* (${{\rm{F}}}_{{\rm{e}}}$) were calculated from the following equations^2^

7 $${{\rm{F}}}_{{\rm{y}}}=2{{\rm{\pi }}r}_{{\rm{y}}}^{3}{{\rm{\varepsilon }}}_{0}{\rm{real}}({{\rm{\varepsilon }}}_{{\rm{r}},{\rm{f}}}^{\ast }){\rm{real}}({{\rm{K}}}_{{\rm{y}}})\nabla {|{\rm{E}}|}^{2}$$

8 $${{\rm{F}}}_{{\rm{e}}}=2{{\rm{\pi }}r}_{{\rm{e}}}^{3}{{\rm{\varepsilon }}}_{0}{\rm{real}}({{\rm{\varepsilon }}}_{{\rm{r}},{\rm{f}}}^{\ast }){\rm{real}}({{\rm{K}}}_{{\rm{e}}})\nabla {|{\rm{E}}|}^{2}$$

where ${r}_{y}$ and ${r}_{e}$ are the radii of yeast and *E. coli*, ${\varepsilon }_{0}\,$is the vacuum permittivity, and ${\varepsilon }_{r,f}^{\ast }$ is the complex relative permittivity of surrounding fluid and was calculated from the following equation:

9 $${{\rm{\varepsilon }}}_{{\rm{r}},{\rm{x}}}^{\ast }={{\rm{\varepsilon }}}_{{\rm{r}},{\rm{x}}}-\frac{i{{\rm{\sigma }}}_{{\rm{x}}}}{{\rm{\omega }}}({\rm{x}}={\rm{f}},\,{\rm{y}},\,{\rm{e}},\,{\rm{s}}1\_{\rm{y}},\,{\rm{s}}2\_{\rm{y}},\,{\rm{s}}1\_{\rm{e}},\,{\rm{s}}2\_{\rm{e}},\,{\rm{s}}3\_{\rm{e}})$$

${\varepsilon }_{r,f}$ is the relative permittivity of fluid.$\,{\sigma }_{f}$ is the conductivity of fluid. ${\varepsilon }_{r,y}$ and ${\varepsilon }_{r,e}$ are the relative permittivity of yeast’s cytoplasm and *E. coli*’s cytoplasm. ${\varepsilon }_{r,s1\_y}$, ${\varepsilon }_{r,s2\_y}\,$are the relative permittivity of the first shell and second shell outside the cytoplasm of yeast.$\,{\varepsilon }_{r,s1\_e}$, ${\varepsilon }_{r,s2\_e}$, ${\varepsilon }_{r,s3\_e}$ are the relative permittivity of first shell, second shell, and third shell outside the cytoplasm of *E. coli*. ${\sigma }_{s1\_y}$, ${\sigma }_{s2\_y}$ are the first shell and second shell outside the cytoplasm. ${\sigma }_{s1\_e}$, ${\sigma }_{s2\_e}$, ${\sigma }_{s3\_e}$ are the conductivity of first shell, second shell, and third shell outside the cytoplasm of *E. coli*. ${K}_{y}$ and ${K}_{e}$ are the Clausius-Mossotti factor of yeast and *E. coli* and were calculated from the following equations.

10 $${{\rm{K}}}_{{\rm{y}}}=\frac{{{\rm{\varepsilon }}}_{{\rm{r}},\text{eq}\_{\rm{s}}2\_{\rm{y}}}^{\ast }-{{\rm{\varepsilon }}}_{{\rm{r}},{\rm{f}}}^{\ast }}{{{\rm{\varepsilon }}}_{{\rm{r}},\text{eq}\_{\rm{s}}2\_{\rm{y}}}^{\ast }+2{{\rm{\varepsilon }}}_{{\rm{r}},{\rm{f}}}^{\ast }}$$

11 $${{\rm{\varepsilon }}}_{{\rm{r}},\text{eq}\_{\rm{s}}2\_{\rm{y}}}^{\ast }={{\rm{\varepsilon }}}_{{\rm{r}},{\rm{s}}2\_{\rm{y}}}^{\ast }\frac{{(\frac{{{\rm{r}}}_{{\rm{o}}\_{\rm{s}}2\_{\rm{y}}}}{{{\rm{r}}}_{{\rm{i}}\_{\rm{s}}2\_{\rm{y}}}})}^{3}+2(\frac{{{\rm{\varepsilon }}}_{{\rm{r}},\text{eq}\_{\rm{s}}1\_{\rm{y}}}^{\ast }-{{\rm{\varepsilon }}}_{{\rm{r}},{\rm{s}}2\_{\rm{y}}}^{\ast }}{{{\rm{\varepsilon }}}_{{\rm{r}},\text{eq}\_{\rm{s}}1\_{\rm{y}}}^{\ast }+2{{\rm{\varepsilon }}}_{{\rm{r}},{\rm{s}}2\_{\rm{y}}}^{\ast }})}{{(\frac{{{\rm{r}}}_{{\rm{o}}\_{\rm{s}}2\_{\rm{y}}}}{{{\rm{r}}}_{{\rm{i}}\_{\rm{s}}2\_{\rm{y}}}})}^{3}-(\frac{{{\rm{\varepsilon }}}_{{\rm{r}},\text{eq}\_{\rm{s}}1\_{\rm{y}}}^{\ast }-{{\rm{\varepsilon }}}_{{\rm{r}},{\rm{s}}2\_{\rm{y}}}^{\ast }}{{{\rm{\varepsilon }}}_{{\rm{r}},\text{eq}\_{\rm{s}}1\_{\rm{y}}}^{\ast }+2{{\rm{\varepsilon }}}_{{\rm{r}},{\rm{s}}2\_{\rm{y}}}^{\ast }})}$$

12 $${{\rm{\varepsilon }}}_{{\rm{r}},\text{eq}\_{\rm{s}}1\_{\rm{y}}}^{\ast }={{\rm{\varepsilon }}}_{{\rm{r}},{\rm{s}}1\_{\rm{y}}}^{\ast }\frac{{(\frac{{{\rm{r}}}_{{\rm{o}}\_{\rm{s}}1\_{\rm{y}}}}{{{\rm{r}}}_{{\rm{i}}\_{\rm{s}}1\_{\rm{y}}}})}^{3}+2(\frac{{{\rm{\varepsilon }}}_{{\rm{r}},{\rm{y}}}-{{\rm{\varepsilon }}}_{{\rm{r}},{\rm{s}}1\_{\rm{y}}}^{\ast }}{{{\rm{\varepsilon }}}_{{\rm{r}},{\rm{y}}}+2{{\rm{\varepsilon }}}_{{\rm{r}},{\rm{s}}1\_{\rm{y}}}^{\ast }})}{{(\frac{{{\rm{r}}}_{{\rm{o}}\_{\rm{s}}1\_{\rm{y}}}}{{{\rm{r}}}_{{\rm{i}}\_{\rm{s}}1\_{\rm{y}}}})}^{3}-(\frac{{{\rm{\varepsilon }}}_{{\rm{r}},{\rm{y}}}-{{\rm{\varepsilon }}}_{{\rm{r}},{\rm{s}}1\_{\rm{y}}}^{\ast }}{{{\rm{\varepsilon }}}_{{\rm{r}},{\rm{y}}}+2{{\rm{\varepsilon }}}_{{\rm{r}},{\rm{s}}1\_{\rm{y}}}^{\ast }})}$$

13 $${{\rm{K}}}_{{\rm{e}}}=\frac{{{\rm{\varepsilon }}}_{{\rm{r}},\text{eq}\_{\rm{s}}3\_{\rm{e}}}^{\ast }-{{\rm{\varepsilon }}}_{{\rm{r}},{\rm{f}}}^{\ast }}{{{\rm{\varepsilon }}}_{{\rm{r}},\text{eq}\_{\rm{s}}3\_{\rm{e}}}^{\ast }+2{{\rm{\varepsilon }}}_{{\rm{r}},{\rm{f}}}^{\ast }}$$

14 $${{\rm{\varepsilon }}}_{{\rm{r}},\text{eq}\_{\rm{s}}3\_{\rm{e}}}^{\ast }={{\rm{\varepsilon }}}_{{\rm{r}},{\rm{s}}3\_{\rm{e}}}^{\ast }\frac{{(\frac{{{\rm{r}}}_{{\rm{o}}\_{\rm{s}}3\_{\rm{e}}}}{{{\rm{r}}}_{{\rm{i}}\_{\rm{s}}3\_{\rm{e}}}})}^{3}+2(\frac{{{\rm{\varepsilon }}}_{{\rm{r}},\text{eq}\_{\rm{s}}2\_{\rm{e}}}^{\ast }-{{\rm{\varepsilon }}}_{{\rm{r}},{\rm{s}}3\_{\rm{e}}}^{\ast }}{{{\rm{\varepsilon }}}_{{\rm{r}},\text{eq}\_{\rm{s}}2\_{\rm{e}}}^{\ast }+2{{\rm{\varepsilon }}}_{{\rm{r}},{\rm{s}}3\_{\rm{e}}}^{\ast }})}{{(\frac{{{\rm{r}}}_{{\rm{o}}\_{\rm{s}}3\_{\rm{e}}}}{{{\rm{r}}}_{{\rm{i}}\_{\rm{s}}3\_{\rm{e}}}})}^{3}-(\frac{{{\rm{\varepsilon }}}_{{\rm{r}},\text{eq}\_{\rm{s}}2\_{\rm{e}}}^{\ast }-{{\rm{\varepsilon }}}_{{\rm{r}},{\rm{s}}3\_{\rm{e}}}^{\ast }}{{{\rm{\varepsilon }}}_{{\rm{r}},\text{eq}\_{\rm{s}}2\_{\rm{e}}}^{\ast }+2{{\rm{\varepsilon }}}_{{\rm{r}},{\rm{s}}3\_{\rm{e}}}^{\ast }})}$$

15 $${{\rm{\varepsilon }}}_{{\rm{r}},\text{eq}\_{\rm{s}}2}^{\ast }={{\rm{\varepsilon }}}_{{\rm{r}},{\rm{s}}2}^{\ast }\frac{{(\frac{{{\rm{r}}}_{{\rm{o}}\_{\rm{s}}2\_{\rm{e}}}}{{{\rm{r}}}_{{\rm{i}}\_{\rm{s}}2\_{\rm{e}}}})}^{3}+2(\frac{{{\rm{\varepsilon }}}_{{\rm{r}},\text{eq}\_{\rm{s}}1\_{\rm{e}}}^{\ast }-{{\rm{\varepsilon }}}_{{\rm{r}},{\rm{s}}2\_{\rm{e}}}^{\ast }}{{{\rm{\varepsilon }}}_{{\rm{r}},\text{eq}\_{\rm{s}}1\_{\rm{e}}}^{\ast }+2{{\rm{\varepsilon }}}_{{\rm{r}},{\rm{s}}2\_{\rm{e}}}^{\ast }})}{{(\frac{{{\rm{r}}}_{{\rm{o}}\_{\rm{s}}2\_{\rm{e}}}}{{{\rm{r}}}_{{\rm{i}}\_{\rm{s}}2\_{\rm{e}}}})}^{3}-(\frac{{{\rm{\varepsilon }}}_{{\rm{r}},\text{eq}\_{\rm{s}}1\_{\rm{e}}}^{\ast }-{{\rm{\varepsilon }}}_{{\rm{r}},{\rm{s}}2\_{\rm{e}}}^{\ast }}{{{\rm{\varepsilon }}}_{{\rm{r}},\text{eq}\_{\rm{s}}1\_{\rm{e}}}^{\ast }+2{{\rm{\varepsilon }}}_{{\rm{r}},{\rm{s}}2\_{\rm{e}}}^{\ast }})}$$

16 $${{\rm{\varepsilon }}}_{{\rm{r}},\text{eq}\_{\rm{s}}1\_{\rm{e}}}^{\ast }={{\rm{\varepsilon }}}_{{\rm{r}},{\rm{s}}1\_{\rm{e}}}^{\ast }\frac{{(\frac{{{\rm{r}}}_{o\_{\rm{s}}1\_{\rm{e}}}}{{{\rm{r}}}_{{\rm{i}}\_{\rm{s}}1\_{\rm{e}}}})}^{3}+2(\frac{{{\rm{\varepsilon }}}_{{\rm{r}},{\rm{e}}}-{{\rm{\varepsilon }}}_{{\rm{r}},{\rm{s}}1\_{\rm{e}}}^{\ast }}{{{\rm{\varepsilon }}}_{{\rm{r}},{\rm{e}}}+2{{\rm{\varepsilon }}}_{{\rm{r}},{\rm{s}}1\_{\rm{e}}}^{\ast }})}{{(\frac{{{\rm{r}}}_{{\rm{o}}\_{\rm{s}}1\_{\rm{e}}}}{{{\rm{r}}}_{{\rm{i}}\_{\rm{s}}1\_{\rm{e}}}})}^{3}-(\frac{{{\rm{\varepsilon }}}_{{\rm{r}},{\rm{e}}}-{{\rm{\varepsilon }}}_{{\rm{r}},{\rm{s}}1\_{\rm{e}}}^{\ast }}{{{\rm{\varepsilon }}}_{{\rm{r}},{\rm{e}}}+2{{\rm{\varepsilon }}}_{{\rm{r}},{\rm{s}}1\_{\rm{e}}}^{\ast }})}$$

${r}_{o\_x}$ and ${r}_{i\_x}$ (x = s1_y, s2_y, s1_e, s2_e, s3_e) are the outer radius and inner radius of the shell.

Drag force also acts on the cell in solution and this is given by:

17 $${\rm{F}}=\frac{1}{{{\rm{\tau }}}_{{\rm{p}}}}{{\rm{m}}}_{{\rm{p}}}({\rm{u}}-{\rm{v}})$$

18 $${{\rm{\tau }}}_{{\rm{p}}}=\frac{{{\rm{\rho }}}_{{\rm{p}}}{{\rm{d}}}_{{\rm{p}}}^{2}}{18{\rm{\mu }}}$$

The solution to this set of equations was obtained using a Finite Element Model (FEM) implemented in COMSOL Multiphysics 4.3. A triangular mesh was used in the numerical analysis (Fig. 1c). Several concentric semi-circles were added near the gap between the electrodes (NEME) to yield a higher resolution in the regions in which the expected electric potential had higher gradients (See Fig. 1c). We solved simultaneously for the electric field and the fluid flow. Particle tracking was used to follow each cell as it moves due to the combined forces of DEP and drag force throughout the electroporation process. The time step employed was 0.001 s and mesh refining was used to evaluate the convergence of the numerical calculations.

### Data availability

The datasets generated during and/or analyzed during the current study are available from the corresponding author on reasonable request.

## Electronic supplementary material

Supplementary figure S1
