# Supplementary material for: Simultaneous electroporation and dielectrophoresis in non-electrolytic micro/nano-electroporation
Source: Sci Rep. 2018 Feb 6;8:2481. doi: 10.1038/s41598-018-20535-6 (PMC5802840; doi:10.1038/s41598-018-20535-6)

Simultaneous electroporation and dielectrophoresis in non-electrolytic micro/nano-electroporation

Chenang Lyu* ^1^, ^2^, Jianping Wang^1^, Matthew Powell-Palm^2^, Boris Rubinsky^2^

**Supplementary**

**Figure S1** The mesh (triangle) distribution without the concentric rings near the gap.

Figure S1


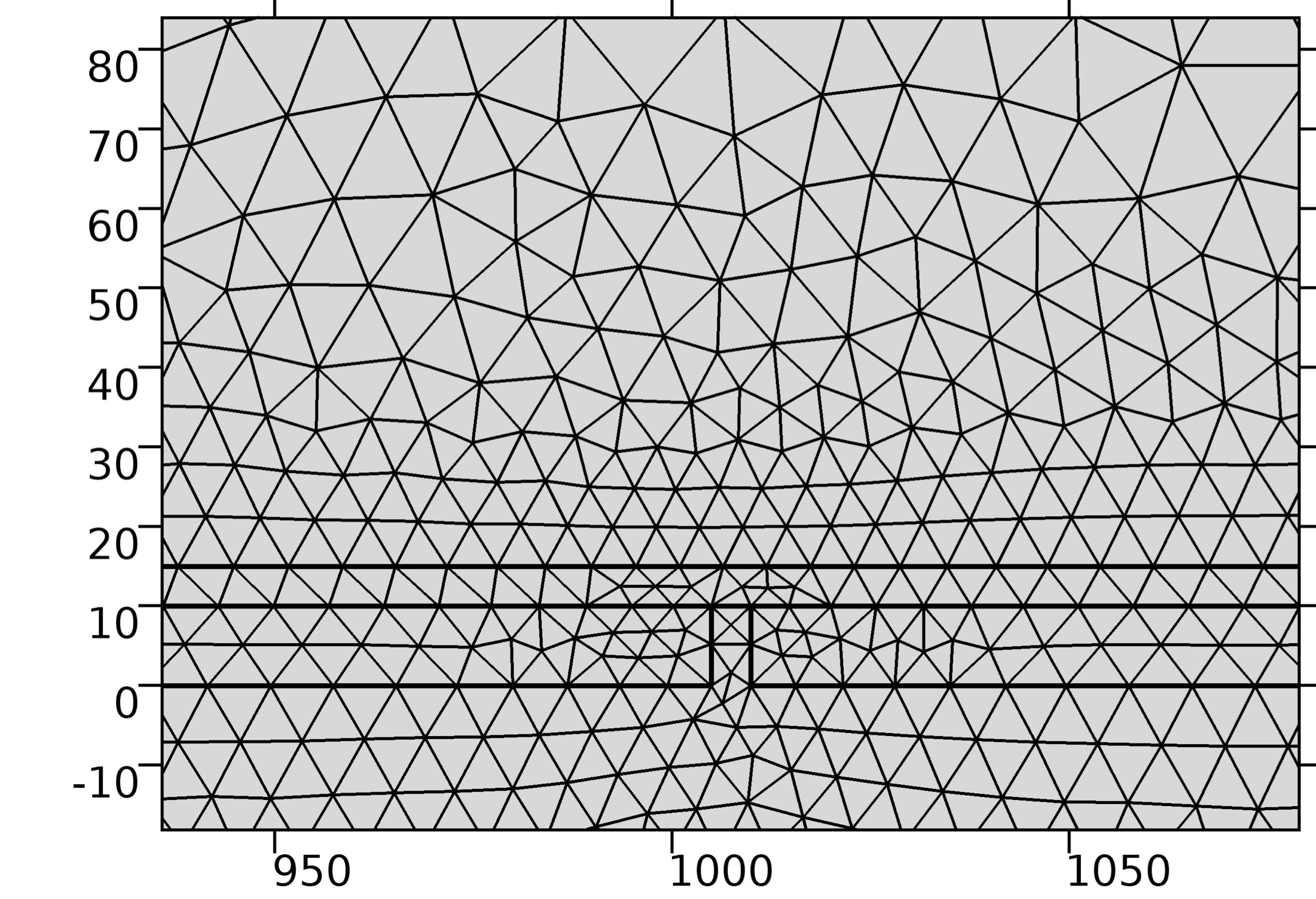

Supplement: Supplementary file 1 — Supplementary figure S1 [file 41598_2018_20535_MOESM1_ESM.doc]
